# Unraveling Vulnerabilities and Pathways Responsible for AITL Malignancy to Improve Patients’ Treatment

**DOI:** 10.3390/pharmaceutics18101292

**Published:** 2026-10-12

**Authors:** Chiara Martinello, Adrien Krug, Pau Marin-Escudero, Aurélia Pollet, François Lemonnier, Laurent Boyer, Els Verhoeyen

**Affiliations:** 1 Université Côte d’Azur, Institut National de la Santé et de la Recherche Médicale (INSERM), C3M, 06204 Nice, France; chiara.martinello@etu.univ-cotedazur.fr (C.M.); adrien.krug@etu.univ-cotedazur.fr (A.K.); aurelia.pollet@univ-cotedazur.fr (A.P.); laurent.boyer@univ-cotedazur.fr (L.B.); 2 Laboratory of Advanced Genome Editing Technologies, Department Centre for Integrative Biology (CIBIO), University of Trento, 38122 Trento, Italy; pau.marinescudero@unitn.it; 3 INSERMU955, Unité Hémopathies Lymphoïdes, Institut Mondor de Recherche Biomédicale, Université Paris-Est Créteil, Hôpitaux Universitaires Henri Mondor, Assistance Publique des Hôpitaux de Paris, 94000 Créteil, France; francois.lemonnier@aphp.fr; 4 CIRI—International Center for Infectiology Research, Inserm, U1111, Centre National de la Recherche Scientifique (CNRS), UMR5308, Ecole Normale Supérieure de Lyon, Université Claude Bernard Lyon 1, Université Lyon, 69007 Lyon, France

**Keywords:** AITL, T-cell lymphoma, epigenetics, immunotherapy, CAR T, metabolism, AITL preclinical mouse model, RhoA, Tet2, T follicular helper cell

## Abstract

Angioimmunoblastic T-cell lymphoma (AITL) is an aggressive subtype of peripheral T-cell lymphoma characterized by poor clinical outcomes following conventional CHOP-based chemotherapy and the lack of effective disease-specific therapeutic options. The disease finds its origin in CD4 follicular helper T (Tfh) cells and is driven by recurrent genetic alterations, including mutations in *TET2*, *RHOA*, *DNMT3A*, and *IDH2.* Despite the rarity of AITL, preclinical models have been fundamental in elucidating disease pathogenesis, identifying key molecular vulnerabilities and providing platforms for the evaluation of novel therapeutic strategies. These studies have highlighted multiple targetable pathways, including aberrant mTOR, T-cell receptor and NF-κB signaling. Additionally, widespread epigenetic alterations in the neoplastic T-cells were uncovered due to mutations in epigenetic modifiers in AITL. More recently, metabolic reprogramming has emerged as an additional therapeutic vulnerability, revealing a strong dependence of AITL cells on mitochondrial respiration and lipid metabolism, particularly the choline metabolic pathway. Advances in the understanding of AITL biology have also accelerated the development of immunotherapeutic strategies. Monoclonal antibodies, antibody–drug conjugates, bispecific antibodies, immune checkpoint inhibitors and chimeric antigen receptor T-cell therapies have shown encouraging preclinical and early clinical activity. Of note, the first preclinical results on an in vivo CAR-T-cell approach for AITL are reported. Here we provide a broad overview of AITL, discussing its biological features, pathogenesis, preclinical models, current treatment landscape, and recent advances in therapeutic approaches.

## 1. The Complex Microenvironment of Angioimmunoblastic T-Cell Lymphoma

Peripheral T-cell lymphomas (PTCLs) represent 12–15% of all lymphoid malignancies in Western Europe and account for even more cases in Japan [1]. The most frequent ones are called PTCL NOS (not otherwise specified), anaplastic large-cell lymphoma and angioimmunoblastic T-cell lymphoma (AITL). Nevertheless, T-cell lymphomas are rare as compared to B-cell lymphomas. Importantly, in 2016 the World Health Organization (WHO) revised the T-cell lymphoma classification, in which AITL, follicular T-cell lymphoma, and nodal PTCL with Tfh phenotype were unified in a single classification named hereafter Tfh-like PTCL [2]. The overall 5-year survival rates for AITL are estimated at 30–40% with conventional (CHOP) chemotherapy, and at the moment, no validated disease-specific effective therapies are available [3,4,5]. One problem is that AITL reveals itself only at advanced age, with patients developing splenomegaly, hepatomegaly, enlarged lymph nodes marked by a completely aberrant architecture and frequently bone marrow (BM) and extra-lymphatic involvement in the liver, skin, gastrointestinal tract, and lungs [6,7,8,9]. Further clinical signs are fever, sweating, weight loss, inflammation due to severe immune dysregulation (high levels of autoimmune antibodies), skin rash and ascites. The drivers of AITL are CD4 follicular helper T-cells (Tfh) expressing Tfh-specific surface molecules such as the checkpoint inhibitor PD-1, inducible co-stimulatory (ICOS), CD10, B-cell lymphoma 6 (Bcl-6), FAS and chemokine receptors such as CXCR5 [10,11,12,13,14,15,16]. The clonal nature of these cells, evaluated by T-cell receptor (TCR) analysis, confirmed that the Tfh cells in AITL were neoplastic. They are dysregulated in growth and begin to release chemokines and cytokines (IL-6, CCL17, IL-21, CXCL13, and VEGF1; [13,15]) to regulate a network of interactions with a complex tumor microenvironment (TME). CXCL13 is secreted by the Tfh cells and binds to the CXCR5 receptor, inducing B-cell and follicular dendritic cell (FDC) accumulation. Therefore, the TME consists of germinal center (GC) and plasma B-cells, follicular dendritic cells, as well as strongly suppressed/exhausted macrophages and effector T-cells [17,18]. The level of infiltrating CD8 TILs (tumor-infiltrating lymphocytes) correlates with the extent of exhaustion of these T-cells, characterized by high-level expression of immune checkpoints (IC) [19]. This high proportion of exhausted CD8 TILs in AITL tumors is a significant risk factor for overall survival (OS) [20]. Though the neoplastic T-cells and their microenvironment are now well-known, diagnosis remains often difficult, and detection occurs at a late stage of disease because these malignant cells seldom circulate in the peripheral blood of the patients.

## 2. Mutational Landscape in AITL

Though AITL is a complex disease, genomic analysis highlights a relatively homogeneous mutational landscape in which ten-eleven translocation-2 (*TET2*) mutations are present most frequently in these patients (80%), followed by Ras homolog family member A (*RHOA*) mutations (50–70% of patients) [21,22]. In addition, *DNMT3A* and isocitrate dehydrogenase-2 (*IDH2*) mutations are detected in 20–30% of AITL patients [23,24]. *TET2* is a tumor suppressor gene coding for an a-ketoglutarate (aKG) dioxygenase, inducing 5-hydroxymethyl cytosine formation. DNMT3A is a DNA methyltransferase that converts 5-cytosine to 5-methyl cytosine while RHOA, a GTPase, is essential in T-cell migration and function. The *RHOA*^G17V^ mutation is the most common one detected in AITL [21,25,26]. *IDH2* mutations (most frequently *IDH2^R172^*) result in a new enzyme activity that reduces a-ketoglutarate (aKG) to D-2- hydroxyglutarate (D2HG) [24]. D2HG can inhibit several dioxygenases such as histone demethylases and TET proteins involved in DNA demethylation that use aKG as a cofactor. Importantly, multiple other gene mutations are implicated in TCR signaling pathways (i.e., *CD28*, *RHOA*, *VAV1* and *FYN*, *PLCG1*) [26,27,28,29,30,31]. As expected, these disturb TCR activation or co-stimulatory pathways in AITL, though at lower frequency [32]. Several reports suggested that AITL develops stepwise, with *TET2* and *DNMT3A* mutations first appearing in hematopoietic progenitors leading to clonal hematopoiesis, followed by ‘‘second hits’’ such as *RHOA* and *IDH2* mutations that drive the transformation into neoplastic CD4 Tfh-like cells [33,34].

Therefore, it made perfect sense to combine *TET2*, *RHOA*, *DNMT3A* and *IDH2* mutations found in patients and in AITL in mouse models, which resulted in preclinical models for PTCL/AITL-like disease as detailed below.

## 3. Preclinical Mouse Models Recapitulating AITL Disease: Powerful Tools for Discovery of New Therapies

**Figure 1:**
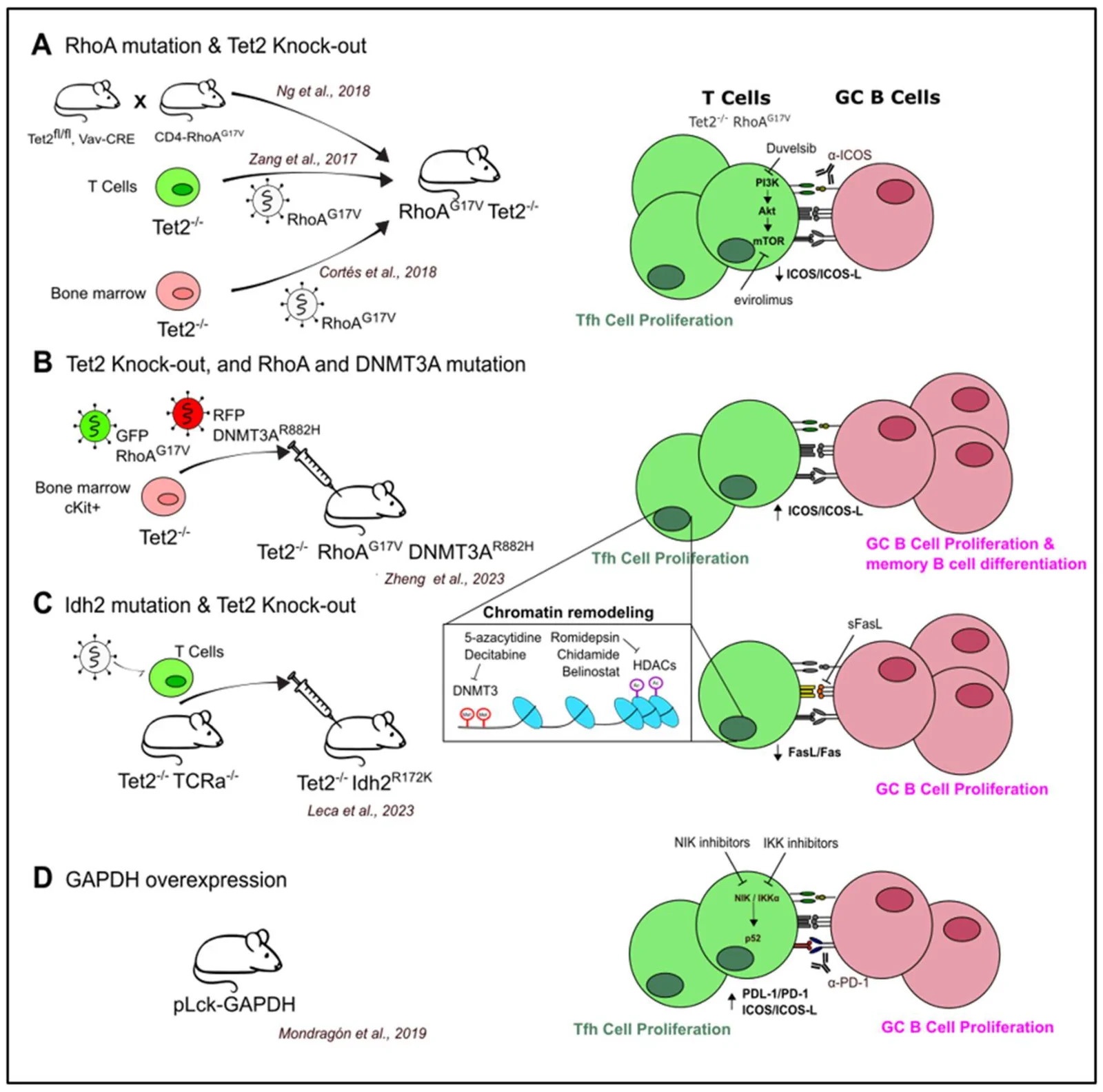
**Genetic preclinical mouse models mimicking human AITL disease.** (**A**) AITL mouse models based on *Tet2* knockout and introduction of *RhoA^G17V^* mutation by (1) crossing a mouse with *Tet* KO in all hematopoietic cells with a mouse carrying *RhoA^G17V^* mutation in CD4 T-cells [38]; (2) Transfer of *Tet2*^−/−^ T-cells transduced with a retroviral vector encoding *Rho^G17V^* into wt mice [36]; or (3) transfer of *Tet2*^−/−^ BM cells transduced with a retroviral vector encoding *RhoA^G17V^* into wt mice [37]. (**B**) AITL mouse model based on introduction of *RhoA^G17V^* mutations and a *Dnmt3a* mutation by transduction of Tet2^−/−^ bone marrow cKit+ cells with two different vectors encoding *RhoA^G17V^* or *Dnmt3a^R882H^*, which were then transferred into wt mice [23]. (**C**) AITL mouse model relying on transfer of *Tet2*^−/−^ T-cells that were transduced with a retroviral vector encoding *Idh^2R172K^* into wt mice [41]. (**D**) AITL mouse model based on a transgenic mouse overexpressing GAPDH exclusively in T-cells [42]. On the right are shown the different pathways activated in the Tfh-like AITL T-cells or surface receptor interactions of CD4 Tfh cells (in green) with the GC B-cells (in pink) in the lymphoma microenvironment. Additionally, some therapeutic interventions are indicated.

From 2005 onwards, several AITL-like mouse models were developed, such as the Roquin^san^ and Swiss Jim Lambert mouse models, as well as two models that were based on single-gene mutations, recurrently found in AITL patients: the single mutant *Tet2*^−/−^ mice, *Idh2^R17K^* and *RhoA^G17V^* mice [35]. These models, though, only partially recapitulate this peripheral T-cell lymphoma. As mentioned above, mutations in *TET2*, *IDH2*, *RHOA* and *DMT3A* are detected in combinations in AITL patients. This led to the generation of three models based on *Tet2* invalidation combined with overexpression of the *RhoA^G17V^* mutation (Figure 1A). A first model by Zang et al. [36] extracted T-cells from *Tet2*^−/−^ mice and transduced them with a retroviral vector allowing *RhoA^G17V^* overexpression in the context of *Tet2* invalidation in these T-cells. These were then transferred to recipient mice that developed a T-cell lymphoma with strongly proliferating CD4 Tfh cells (PD-1^high^, CXCR5^+^, ICOS^+^). The inactivation of TET2 and RHOA^G17V^ overexpression affected genes implicated in T-cell differentiation. These mice suffered from acute inflammation and sudden death, making it hard to achieve transformation. To generate a second mouse model, Cortes et al. isolated BM from *Tet2*^−/−^ mice and transduced them with a retroviral vector encoding *RhoA^G17V^*, followed by engraftment into recipient mice (Figure 1A,C, [37]). These mice developed lymphoma with evident features defining AITL: enlarged spleen and lymph nodes containing monoclonal Tfh cells carrying an AITL gene expression signature. A third model consists of a transgenic mouse expressing RHOA^G17V^ under the control of murine CD4 regulatory elements (CD4-*RhoA^G17V^*), which was crossed with *Tet2*^−/−^ mice harboring a deleted *Tet2* in all hematopoietic lineages [38]. These mice were further crossed with OT-II mice and immunized with ovalbumin to mimic chronic T-cell activation, upon which they developed enlarged spleens and lymph nodes, marked by a pronounced increase in clonal CD4 Tfh cells. As in the previous studies, the combination of *RhoA^G17V^* and *Tet2* loss-of-function mutations gave rise to Tfh-like lymphoma similar to hAITL. Importantly, these three studies underlined the importance of *Tet2* in T-cell epigenetic alterations, while *RhoA* mutation activated TCR signaling, T-cell proliferation and their differentiation into Tfh cells (Figure 1A). A more detailed side-by-side comparison of these three *RhoA^G17V^*/*Tet2*^−/−^ models is reported elsewhere [35].

Of note, together with *RHOA* and *TET2* mutations, mutations in the DNA methyl transferase, *DNMT3A*, were detected in 28% of the AITL patients [21,22]. *DNMT3A^R882H^* is the most common mutation in AITL and was shown to have a cooperative effect with *TET2* loss in promoting T-cell lymphoma. Zheng et al. [23] wanted to evaluate the role of *Dnmt3a^R882H^* in AITL development when co-occurring with *RhoA^G17V^* and *Tet2* loss. They isolated hematopoietic stem cells from the bone marrow of *Tet2*^−/−^ mice and transduced them with two retroviral vectors, one encoding for RHOA^G17V^ and GFP and another vector coding for DNMT3A^R882H^ and mCherry, which allowed them to follow the fate of triple mutant GFP+ mCherry+ cells upon injection into congenic recipient mice (Figure 1B). These *Dnmt3a^R882H^*, *RhoA^G17V^*, and *Tet2*^−/−^ mice developed AITL-like symptoms such as skin rash, severe inflammation and aberrant follicular structure in the enlarged spleen and LN. The CD4 Tfh (PD-1^high^, BCL6^+^ and CXCR5^+^) population was increased, and these neoplastic cells were monoclonal, equivalent to human Tfh cells in AITL. The addition of the mutated Dnmt3a^R882H^ accelerated the development of the T-cell lymphoma by enhancing expansion and hyperactivity of the malignant Tfh cells. It also led to strong interactions of Tfh cells with the B-cells in the lymphoma microenvironment via ICOS-ICOSL, PD-1-PDL1, and CD28-CD86, resulting in abnormal B-cell proliferation and maturation into memory B-cells.

Since mutations in *IDH2* and *TET2* also frequently co-occur in AITL disease [39,40], a genetic mouse model for AITL was generated combining *Idh2^R172K^* and *Tet2* KO mutations, specifically in mouse T-cells. After adoptive T-cell transfer into TCR knock-out mice, this *Idh2^R172K^*/*Tet2*^−/−^ mouse model mimicked AITL disease, and the combination of these two mutations altered Tfh cell function at the epigenetic and gene expression level (Figure 1C, [41]). These mice possessed monoclonal or oligoclonal T-cell populations accompanied by elevated levels of GC B-cells. The lymphoma is marked by a perturbed architecture of the LNs and spleen, branching blood vessels, and infiltration of CD4 T-cells. GC B-cell and plasma cell numbers in these hematopoietic tissues increased with AITL disease progression, sometimes accompanied by clonal expansion of B-cells or plasma cells. In accordance, the authors suggest that *Idh2* and *Tet2* mutations collaborate to reprogram the Tfh cell gene expression and cytokine profiles, likely modulating Tfh cell functions and interactions with other components of the TME, in particular with B-cells. Given the expected effects of *Idh2* and *Tet2* mutations on epigenetics, hypermethylation of specific promoters led to altered gene expression in *Idh2^R172K^; Tet2*^−/−^ Tfh cells (e.g., chemokines, inflammatory responses, transcription factors, TCR signaling, and angiogenesis). These malignant *Idh2^R172K^; Tet2*^−/−^ Tfh cells not only increase the proliferation and differentiation of B-cells but also boost their survival by weakening Fas-Fas ligand interaction and downregulating Fas-mediated apoptosis. Moreover, competition for binding to Fas with a soluble Fas ligand resulted in B-cell death (Figure 1C). Since this *Idh2*/*Tet2* model is missing the T-cell activating component, it might be of great interest to generate a triple transgenic mouse, *Idh2 ^R172K^*/*Tet2*^−/−^/*RhoA^G17V^*, that might even better recapitulate the phenotype and malignancy of a subgroup of AITL patients.

Finally, another AITL preclinical model was developed by genetically overexpressing exclusively in T-cells glyceraldehyde 3-phosphate dehydrogenase (GAPDH), a key glycolytic enzyme in T-cell survival and function ([42], Figure 1D). Surprisingly, these mice developed AITL-like malignancy after 18 months, characterized by enlarged spleens and lymph nodes with aberrant architecture. This T lymphoma was defined by infiltration of equivalent levels of clonal or oligoclonal neoplastic CD4 Tfh cells, GC B-cells and infiltrating follicular dendritic cells, typical components of the TME in AITL. Moreover, all clinical features of AITL were recapitulated in this model: GC differentiation into plasma cells secreting high levels of antibodies, chronic inflammation, skin rash and ascites. Spontaneously, these mice acquired a mutation in *RhoA*; however, not at aa position 17, as frequently detected in human AITL, but at aa position 37 (T37M). Both mutations, though, resulted in loss of binding to GTP, favoring neoplastic T-cell growth. This underlines the importance of *RhoA* mutation again in the uncontrolled proliferation of Tfh neoplastic cells, as shown already above for the *RhoA^G17V^*/*Tet2*^−/−^ models [36,37,38]. Other mutations in the epigenetic modifiers *Tet2*/*Dmnt3A*/*Idh2* were not identified in these AITL mice. In this model, the level of Tfh neoplastic cells is very similar to the accompanying GC B-cells, mutually important for each other’s proliferation and survival.

Due to the rareness of AITL, the available genetic AITL preclinical models offered a precious opportunity to reveal new pathways or vulnerabilities in these tumor cells and evaluate various novel, more targeted treatments, urgently needed for these patients.

## 4. AITL: Possible Treatment Options

### 4.1. First-Line Therapy for AITL: Chemotherapy and Autologous or Allogenic Stem Cell Transplantation

Currently, first-line treatment for AITL/PTCL is a chemotherapy regimen called CHOP, which consists of cyclophosphamide + doxorubicin + vincristine + prednisolone. However, because patients are diagnosed almost exclusively at an advanced stage and due to the aggressiveness of these T-cell lymphomas, treatment is not effective and leads to poor survival outcomes (5-year overall survival 30%) [3,4,5]. Currently, chemotherapy followed by autologous stem-cell transplantation (ASCT) remains the standard of care in first-line or relapsed/refractory settings, in combination or not with etoposide (CHOEP; [43,44]). Adding etoposide to the standard CHOP therapy regimen creates the CHOEP (or CHOPE) chemotherapy, which is mainly used to treat aggressive types of non-Hodgkin lymphoma and PTCL. Etoposide is a topoisomerase II inhibitor that introduces DNA breaks, leading to cancer cell killing. Unfortunately, the addition of etoposide is associated with toxicity due to severe side effects such as myelosuppression, neutropenia and gastrointestinal complications. For these reasons, CHOEP is recommended primarily for younger patients (<65) who are fit enough to endure this harsh treatment, and who mainly suffer from aggressive PTCL. Importantly, salvation ASCT is better supported and efficient in younger patients (<60 years old; [45]). Unfortunately, some data from clinical trials, though showing improved survival, also revealed treatment-related toxicities and therefore do not support this approach.

Alternatively, allogeneic hematopoietic cell transplantation (allo-HCT) is considered a common treatment for T-cell lymphomas. Interestingly, as compared to PTCL-NOS, patients with AITL had a significantly lower risk of relapse and better progression-free survival (PFS). This is in accordance with the fact that AITL appears to be extremely sensitive to graft-versus-leukemia effects. Consistently, patients with AITL showed the most favorable outcomes [44]. Another study showed excellent survival of patients with AITL after allo-HCT (4-year survival, 70%) and no relapses beyond 2 years [46].

Unfortunately, these current treatments are not sufficient or have high risks, and an effective standard therapy still needs to be defined.

### 4.2. Signaling Pathways That Lead the Way to New Therapies

#### 4.2.1. NF-κB Pathway Activation in AITL

In one of the murine AITL mouse models by Mondragon et al. [42] a strong activation of the non-canonical NF-κB pathway was revealed in the malignant Tfh-like cells. One of the first-acting enzymes in this pathway, the NF-κB-inducing Kinase (NIK), plays an important role in B and T helper cell differentiation and development [47]. NIK activates the non-canonical NF-κB pathway through IκB kinase (IKK) phosphorylation. Treatment with a specific inhibitor of NIK [32,42] resulted in reduced lymphoma development and an increased survival rate in the preclinical mAITL mice. Additionally, we need to emphasize that GC B-cells also highly express NIK and that they play a crucial role in the survival of healthy Tfh cells as well as AITL Tfh malignant cells [48,49,50]. Although this NIK inhibitor increased mAITL mouse survival, the treatment was fully optimized when combined with an anti-PD-1 immunotherapy. For the moment, neither a NIK inhibitor alone or its combination with anti-PD-1 has been evaluated for treatment of AITL patients, though this could be of benefit for these patients. NIK is also strongly linked to other proteins of the NF-κB pathway such as IKK encoded by NEMO (subunit of the IκB kinase complex that activates NF-κB), which can suppress NIK levels [51]. TBK1 (TANK-bonding kinase 1) can also cause NIK degradation via its phosphorylation on Ser-862 [52]. Thus, these proteins might be potential therapeutic targets. Of note, Bortezomib, a protease inhibitor able to reduce NF-κB activity, showed encouraging results in the treatment of adult T-cell lymphoma [53]. Clinical trials including a heterogenous PTCL patient population are combining this drug with other therapies (NCT04061772; NCT03547700).

#### 4.2.2. Dysfunction of the TCR Signaling Pathway in AITL

In PTCL and AITL patients, mutations in components of the TCR signaling pathway triggering constitutive activation of the TCR play a major role in T-cell pathogenesis [28,51,54].

*RHOA* mutations, very frequently found in AITL neoplastic T-cells, have a strong effect on T-cell adhesion, migration and proliferation since they are essential for actin polymerization [55]. As indicated above, RhoA mutations in different mouse models drive Tfh cell differentiation [37,38]. Additionally, the *RHOA^G17V^* mutant can also bind vav guanine nucleotide exchange factor 1 (VAV1), causing VAV1 hyperphosphorylation [26] followed by nuclear factor of activated T-cells (NFAT) signaling activation, which in turn induces T-cell proliferation and transformation [30,36,37]. This points to the RHOA-VAV1 axis as a new therapeutic target in AITL [25,30].

TCR-costimulatory molecules such as CD28 provide a secondary signal upon TCR engagement with an antigen, leading to a robust immune response. Importantly, *CD28* is mutated in up to 10% of AITL patients. Two *CD28* mutations (D124 and T195) result in prolonged TCR activation because they possess a stronger affinity for their ligand than the WT CD28, leading to more robust T-cell proliferation [56]. What is clear now is that *CD28* mutations are associated with poor prognosis [54]. Additionally, CD28-ICOS and CD28-CTLA4 gene fusions were found in AITL neoplastic cells [57,58]. The CD28-CTLA4 fusion results in hyperactive CD28 signaling in AITL T-cells [27]. Indeed, CTLA4-CD28 transgenic mice developed an AITL-resembling lymphoma, underlining the CTLA4-CD28 cancer-driving capacity [58]. Using the *Tet2*^−/−^; *RhoA^G17V^* AITL preclinical mouse model, these authors confirmed that dasatinib, a multi-kinase inhibitor, abrogated the hyperactive TCR signaling in these mice, accompanied by their increased survival [59]. This was consolidated in a clinical Phase I/II trial. However, only 5 AITL patients were included, so one needs to be prudent in generalizing these results (UMIN000025856). Importantly, dasatinib also abrogated the RhoA-Vav1 TCR signaling in AITL [26]. As another option, for AITL patients carrying this CTLA4-CD28 fusion, an anti-CTLA4 immunotherapy (ipilimumab) could be proposed [58].

ITK (IL2 Inducible T-Cell Kinase) was found highly phosphorylated in 70% of the AITL patients’ Tfh cells, and this was associated with poor response to chemotherapy treatment [60]. Additionally, Spleen Associated Tyrosine Kinase (SYK), normally only present in B-cells, was detected at aberrantly high levels in a major part of the AITL patients’ Tfh cells [54], suggesting that SYK inhibitors might be effective therapeutic agents. In addition, in some PTCL patients, including AITL, genomic translocations gave rise to ITK-SYK and ITK-FER fusions, which are identified as strong drivers of T-cell lymphoma malignancy [61,62,63]. Therefore, ITK is considered a valid therapeutic target and incites the generation of new candidate drugs. Of note, ITK inhibition leads to a reduction in malignant T-cell invasion and migration [60,64] and might therefore be efficient in ITK+ AITL patients. Additionally, it demonstrated anti-tumor activity in B- and T-cell leukemia in preclinical models [60]. Most probably, a combination of CHOP chemotherapy with an ITK inhibitor might represent a promising treatment. Ibrutinib was also found to inhibit ITK in addition to its original function, the inhibition of BTK (Bruton Tyrosine Kinase) and therefore was evaluated for T-cell lymphoma [65]. Ibrutinib was tested in a clinical trial enrolling T-cell lymphoma patients [66]. However, this resulted only in an overall response rate of 8% in these patients. Currently, Soquelitinib, a new selective ITK inhibitor, is being tested in a phase I clinical trial including patients with refractory T-cell lymphoma, but no distinction between lymphoma subtypes in this trial was provided (NCT03952078).

FYN is a tyrosine kinase that has a pivotal function in T-cell activation. *FYN* mutations in AITL give rise to a constitutively activating mutant, resulting in continuous T-cell activation [28]. Many more mutations in proteins involved in the PI3K or MAPK pathways (e.g., PLCG1, CARD11, CTNNB1) that are implicated in the stabilization of stimulatory signals in T-cells have been reported in AITL. It was actually the NF-κB pathway that was shown to be constitutively upregulated in one of the recent preclinical AITL mouse models [42].

Components or regulators of the JAK/STAT signaling pathway are also found mutated in 27% of PTCL NOS and in 29% of AITL neoplastic T-cells [67]. The JAK/STAT signaling pathway had a negative impact on survival of AITL patients, indicating it as a potential target for this malignancy [68]. This was actually supported by a Phase II clinical trial (NCT02974647; [67]) indicating that ruxolitinib, a JAK 1/2 inhibitor, was effective as a precision therapy across various PTCL subtypes of patients harboring mutations activating the JAK/STAT pathway. Golidocitinib, a JAK1 tyrosine-kinase inhibitor, demonstrated encouraging anti-tumor activity in heavily pre-treated patients with relapsed or refractory PTCL in a phase 1 clinical trial (JACKPOT8 Part A; [69]). In a phase 2 trial (JACKPOT8 Part B) involving 49 centers in Australia, China, South Korea, and the USA, patients with relapsed PTCL or PTCL refractory to other treatments were included NCT04105010; [70]). Golidocitinib showed a favorable benefit–risk profile in these PTCL patients, but further confirmatory studies should be conducted. Cerdulatinib, a drug inhibiting SYK and JAK simultaneously, showed remarkable results in a phase 2a clinical trial in AITL patients and proved to be well tolerated by the patients [71]. Efficacy varied strongly per subtype, demonstrating 51.9% in AITL/Tfh like PTCL cases, but no responses in PTCL-NOS [72]. The results reported with several JAK inhibitors hold promise as a targeted therapy specifically in AITL patients.

### 4.3. Therapeutic Intervention with Epigenetic Modifiers, Which Are Mutated in AITL

Epigenetics is defined as changes in gene expression that can be inherited but without changes in the genetic code of genes [73]. Epigenetic gene regulation consists of DNA methylation and post-translational modifications of histones such as acetylation, methylation, phosphorylation, and ubiquitination [74,75]. It is now common knowledge that cancer induction and progression are directed by multiple genetic and epigenetic modifications that cause either oncogene activation or tumor suppressor gene inactivation. Importantly, due to the reversible nature of epigenetic changes, these can theoretically be reversed by interfering with epigenetic modifiers. Several anti-cancer drugs interfering with epigenome actors have been approved by the FDA and resulted in prolonged survival and reduced toxicity in comparison with conventional chemotherapy [76,77].

#### 4.3.1. Aberrant DNA Methylation and Demethylation in AITL

**Figure 2:**
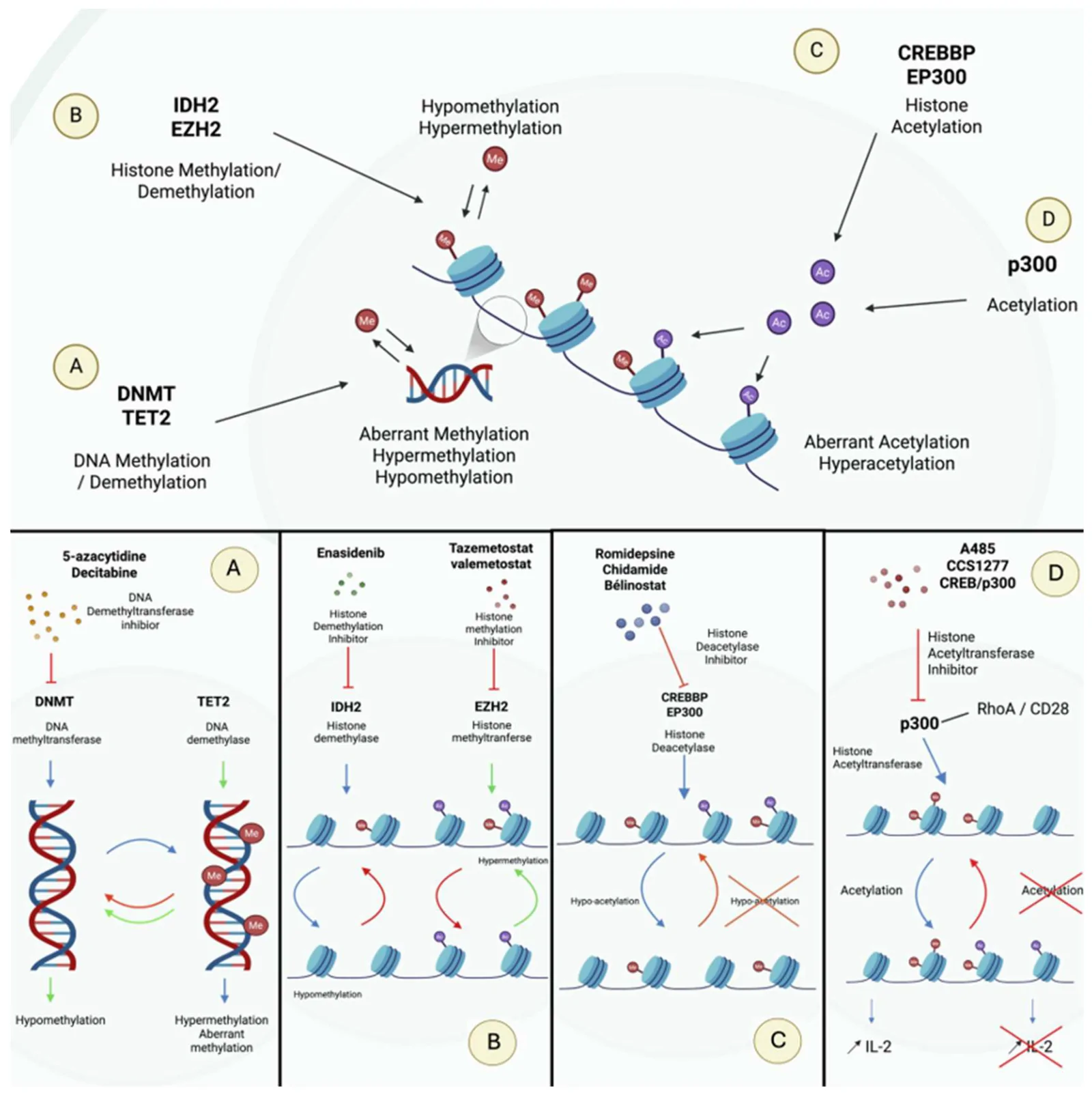
**Interfering with epigenetic modifiers as a novel therapeutic approach in AITL.** Upper scheme showing an overview of the mutated epigenetic modifier genes resulting in modified epigenetic changes as shown in (**A**) for aberrant DNA hypermethylation, (**B**) for histone demethylation, (**C**,**D**) for aberrant acetylation. (**A**) 5-Azacytidine and Decitabine reverts hypermethylation by demethylating DNA; (**B**) Enasidenib is an *IDH2* mutant inhibitor; (**C**) Romidepsine, Chidamide and Belinostat reduce histone hypoacetylation by inhibition of CREBBP and EP300, while Valemostat inhibits EZH2, a histone methyl transferase. (**D**) The drugs A485, CCS1277 and CREB and p300 inhibitors revert aberrant histone acetylation and consequently reduce the high-level secretion of IL-2 in T-cells. Created in BioRender. Legros, V. (2026) https://core.local.biorender.dev/api/short-link/g3d4w5f.

Epigenetic modifications play an essential role in the onset and progression of AITL. This is not surprising since various epigenetic modifying genes such as *TET2*, *IDH2*, *RHOA* and/or *DNMT3A* were reported as mutated in AITL (see point 2). These mutations affect cytosine methylation and hydroxy-methylation. DNA methylation and demethylation regulate gene expression and impact the development and proliferation of cancer cells. Mutations of the *TET2* protein that lead to *TET2* loss of function are associated with hypermethylation of enhancers and promoters in PTCL (Figure 2A). For example, in AITL, dysfunctional TET2 leads to hypermethylation of *BCL6*, resulting in its expression up-regulation, thereby driving Tfh differentiation and T-cell lymphomagenesis [78]. Mutations in *DNMT*s (mainly *DNMT3A* in AITL) can induce aberrant DNA methylation patterns, silencing normally active genes or activating genes that are normally silenced. Therefore, the downstream-regulated pathways are significantly modified, with repercussions on gene transcription, cell differentiation and developmental processes.

Lemonnier and his team highlighted 10 years ago that epigenetic interference treatment with 5-azacytidine can lead to a sustained response in AITL patients (Figure 2A) [79]. Treatment with this hypomethylating agent showed earlier efficacy in myelodysplastic syndrome and acute myeloid leukemia, a response that matches *IDH1*/*2*, *TET2*, and *DNMT3A* mutations detected in these cancers [80,81,82]. Lemonnier included twelve patients with AITL in a clinical trial to evaluate the therapeutic potential of 5-azacytidine. Nine out of twelve patients presented a positive response, six out of twelve a complete response, and three out of twelve a partial response. The overall response rate in this AITL trial was 75%. In all AITL patients, *TET2* mutations were detected, while 33% had additional *DNMT3A* mutations, and 41% had additional *RHOA* mutations. In one single patient, the *IDH2^R172^* mutation was detected together with a *TET2* mutation [79]. The positive outcome of this trial gave way to an international phase III clinical trial (ORACLE: Europe NCT03593018 and Japan NCT03703375) including AITL and Tfh-like PTCL patients, comparing the therapeutic effect and toxicity/safety of oral 5-azacytidine with romidepsin, bendamustine or gemcitabine treatment according to investigator’s choice [83]. The result of the trial confirmed that azacitidine was safe, but the pre-set objectives of efficacy in the trial were not reached for any of these PTCLs. As a single agent, azacitidine was effective in a retrospective study of relapsed/refractory AITL patients, especially when they received less than two chemotherapy treatments [84]. The favorable safety profile indicated that azacitidine could be combined with other drugs such as CHOP. Consistent with this notion, 5-azacytidine was combined with CHOP therapy in a phase II trial for PTCL patients with impressive results (CR was close to 90%) [85]. 5-azacytidine was also used in combination with romidepsin, a histone deacetylase inhibitor, in first-line or relapsing Tfh PTCL patients with an overall response rate of 80%. Together with nivolumab (anti-PD-1), an overall response rate close to 80% in 9 AITL patients was obtained [86,87]. A combination of two epigenetic drugs, 5-azacitidine and chidamide, had the most favorable outcome in a phase 2 study, in particular in AITL [88]. Caution in overinterpretation of these data is warranted since AITL patients represented only a small subgroup in these drug combination trials.

Another agent, decitabine (Figure 2A), a DNA methyltransferase inhibitor that binds to DNA methyltransferases (DNMTs) to induce hypomethylation, has shown promising results in clinical trials. Its combination with an anti-PD-1 immunotherapy demonstrated potential extra benefits for AITL patients due to the extra epigenetic modulation. (NCT03240211) [89].

#### 4.3.2. Modulation of Mutated Histone Markers in AITL as a Therapeutic Option

Histone marks have been discovered for more than half a century, with new mechanisms of action revealed every day. These marks affect gene transcription and chromatin accessibility, with multiple enzymes responsible for inducing or removing these marks [90,91,92]. Mutations in histone markers significantly modify gene regulation and perturb epigenetic regulation [93,94]. The histone marker genes most frequently mutated in AITL and Tfh PTCL are KMT2D (H3K4 methyltransferase), KMT2A (H3K4 methyltransferase), SETD2 (H3K36 methyltransferase), KDM6A (H3K27 demethylase), CREBBP (H3K18 acetyltransferase), EP300 (H3K18 acetyltransferase), and EZH2 (histone methyltransferase) [95,96] (Figure 2).

Romidepsin is a histone deacetylase inhibitor that demonstrated efficacy in relapsed/refractory PTCL [85]. Chidamide is another oral selective HDACi [97] (Figure 2C). A multicenter Phase II trial in China shows increased overall survival in Chidamide-treated AITL patients compared to autologous stem cell transplantation [98]. When Chidamide was combined with CHOP therapy in a phase Ib/II clinical trial for untreated AITL patients, their overall survival was longer than that of AITL patients only treated with CHOP [99]. Similar results were obtained with another HDACi, Belinostat, in a phase II trial for relapsed and refractory PTCL patients and cutaneous T-cell lymphoma (CTCL) (NCT00274651 [100]). In this trial, the number of AITL patients in the PTCL cohort is not disclosed, but PTCL patients reached an ORR that was higher than for CTCL. Recent data suggest that Tfh PTCL could have a better outcome when treated with HDACi than other PTCL subtypes [101]. However, HDACi also induce the deacetylation of non-histone proteins, which can result in unwanted side effects.

Wang et al. [24] provided mechanistic evidence that *IDH2^R172^* mutations alter histone lysine methylation (Figure 2B). Immunohistochemical staining showed significantly elevated H3K27me3 levels (42.2% vs. 16.2% in wild-type), and gene set enrichment analysis (GSEA) further indicated enrichment of H3K27me3-related genes in *IDH2*/*TET2*-double mutants versus *TET2*-single mutants, collectively supporting 2-HG-mediated histone demethylase inhibition in AITL. Enasidenib (AG-221) is a selective mutant *IDH2* inhibitor classified as a histone demethylase inhibitor that specifically targets tumor cells carrying *IDH2* mutations. Enasidenib exerts its therapeutic effect by inhibiting mutant *IDH2*, lowering the high 2-HG levels, and restoring DNA/histone methylation-related processes, while maintaining normal metabolic functions [102,103]. A phase I/II trial targeting *IDH2*-mutated AITL patients and patients with solid tumors carrying the *IDH2* mutation was concluded some time ago; however, results have not been made public (NCT02273739).

In AITL, EZH2 mutations are also often found, which increases the possibility that patients will respond to epigenetic therapy [104]. Furthermore, one patient with TP63 rearrangement in lymphoma showed a good response to valemetostat (EZH2 inhibitor), indicating that some aberrant protein fusions can increase therapeutic vulnerability to EZH2 inhibitors (Figure 2C).

Vallois et al. [105] recently showed that concomitant expression of RHOA^G17V^ and CD28^T195P^ in the context of AITL induced significantly higher levels of interleukin-2 (IL-2) production and nuclear factor of activated T-cells (NFAT) and activator protein 1 (AP1) transcriptional activities than each of these variants alone upon T-cell activation. They identified the histone acetyltransferase p300 as a major interacting partner of RHOA^G17V^ in human primary T-cells. p300-specific H3K18 and H3K27 acetylation marks at the *IL-2* promoter and across the whole genome, respectively, are responsible for this aberrantly high IL-2 secretion in RHOA^G17V^-expressing T-cells. p300 inhibition indeed abolished the increased IL-2 secretion induced by CD3/CD28 stimulation in T-cells harboring both these *RHOA* and *CD28* mutations in Tfh lymphoma (Figure 2D). This suggests that p300 inhibitors may open new therapeutic avenues for Tfh lymphomas such as AITL. Drugs inhibiting p300, such as A485 and CCS1277, were effective in other cellular models of cancer [106,107]. Additionally, a CREB-binding protein/p300 inhibitor is even evaluated in Phase I clinical trials for prostate cancer and carcinoma (NCT04575766 and NCT05488548). For a subset of AITL patients combining *RHOA*^G17V^ and *CD28* mutations, this might provide them with a new therapeutic option but still needs evaluation.

#### 4.3.3. Combining Epigenetic Therapies Is More Successful than Single Agent Treatments

Also, considering the previous chapter of the current review, an important therapeutic strategy relies on the combination of a histone marker inhibitor and a histone deacetylase inhibitor, which resulted in a tighter interaction between KMT2D and PU.1, leading to an inhibition of pERK, which is often upregulated in PTCL. This hypothesis was confirmed in a phase 2 clinical trial combining azacytidine and romidepsin, resulting in significant efficacy in PTCL [86].

Experimental evidence on CD4 T-cells and AITL patients shows that *IDH2^R172^* mutations not only give rise to an increase in 2-HG and hypermethylation of certain promoters but also an augmentation in H3K27me3 marks [101]. Therefore, IDH2^R172^ could represent a potential target for Enasidenib, an *IDH2* mutant inhibitor, or for histone marker agents, as confirmed for acute myeloid leukemia (AML) [108,109]. Nonetheless, these suppositions must be validated in patients.

### 4.4. Immunotherapies on the Horizon to Treat AITL

#### 4.4.1. Antibody-Based Immunotherapy

The complex immunobiology of AITL has progressively shifted therapeutic research toward immune-based strategies. Unlike many other lymphoid malignancies, neoplastic TFH cells reside within a highly dynamic tumor microenvironment composed of immune and stromal cells that actively sustain lymphoma growth, immune dysregulation, and immune evasion [110]. While this intricate cellular network contributes to disease heterogeneity and therapeutic resistance, it also provides a broad spectrum of potential therapeutic targets. Monoclonal antibodies (mAbs) were initially investigated as targeted therapeutic agents in relapsed or refractory (R/R) PTCLs [111]. By selectively targeting surface molecules expressed by malignant T-cells or components of the tumor microenvironment, these agents aim to improve anti-tumor efficacy while limiting the off-target toxicity associated with conventional chemotherapy. Over time, several antibodies have been investigated both as single drugs and in combination with chemotherapy regimens [112].

**Figure 3:**
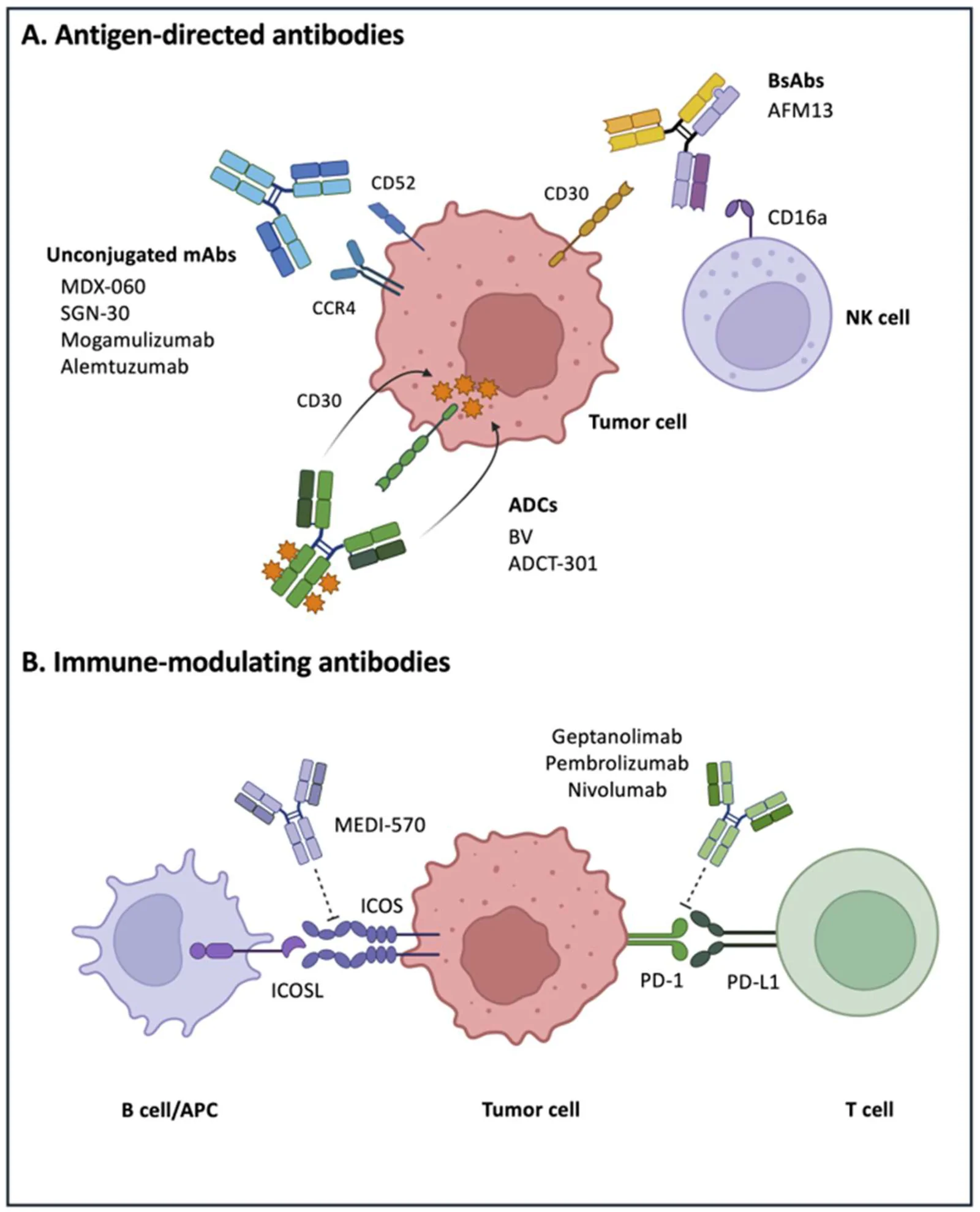
**Antibody-mediated immunotherapy for peripheral T-cell lymphoma.** (**A**) Malignant T-cell antigen-directed antibodies. BsAb: bispecific antibodies, ADC: antibody–drug conjugates, NK: Natural killer, APC: antigen-presenting cell. (**B**). Immune-modulating antibodies. Antibodies interfering with the PDL1-PD-1 immune checkpoint axis between tumor cells and T-cells. Antibodies interfering with the tumor B-cell/APC interaction via ICOS and ICOSL. Created in BioRender. Legros, V. (2026) https://core.local.biorender.dev/api/short-link/fxu4w1z.

Depending on their mechanism of action, antibody-based therapies can be broadly classified into antigen-directed antibodies (Figure 3A), which directly recognize tumor-associated surface antigens and mediate tumor cell elimination, and immune-modulating antibodies (Figure 3B), which restore or enhance anti-tumor immune responses [113]. While some of these approaches have already demonstrated clinical benefit, others remain under active clinical investigation. Importantly, most clinical trials evaluating immunotherapeutic approaches in PTCL were not designed specifically for AITL but enrolled heterogeneous PTCL populations that included a subset of patients with AITL. Therefore, efficacy data should be interpreted with caution, particularly when AITL-specific outcomes were not separately reported. Among antigen-directed approaches, CD30 is the best-established therapeutic target in PTCL [114]. Unconjugated anti-CD30 mAbs, including MDX-060 [115] and SGN-30 [116], demonstrated only modest antitumor activity, highlighting the need for more effective strategies capable of enhancing the cytotoxic potential of antibody-mediated targeting. This led to the development of antibody–drug conjugates (ADCs), which combine the high targeting specificity of monoclonal antibodies with the potent cytotoxic activity of chemotherapeutic payloads [112]. Brentuximab vedotin (BV) is the most successful example of this approach and has been the only antibody-based therapy to significantly improve outcomes in CD30-positive PTCL. BV consists of an anti-CD30 mAbs conjugated to the microtubule-destroying agent monomethyl auristatin E (MMAE). Following antigen binding and internalization, MMAE is released intracellularly, inducing mitotic arrest and apoptosis of the target cell. Early phase II studies in R/R CD30-positive PTCL demonstrated encouraging clinical activity [117], which ultimately led to the phase III ECHELON-2 trial, which compared BV plus cyclophosphamide, doxorubicin and prednisone (A + CHP) with standard CHOP therapy [118]. The study reported significant improvements in both the 5-year PFS and OS (51.4% vs. 43.0%, 70.1% vs. 61.0%, respectively), establishing A + CHP as the standard frontline regimen for patients with CD30-positive PTCL [118,119]. However, the benefit in AITL should be interpreted with caution, as this histological subtype represented only a small proportion of the study population (12%) and subgroup analyses were not powered to detect subtype-specific differences [120]. Interestingly, accumulating evidence suggests that the clinical activity of brentuximab vedotin is not strictly dependent on the level of CD30 expression. Proposed mechanisms include intratumoral antigen heterogeneity, bystander killing mediated by extracellular diffusion of MMAE, and immunomodulatory effects resulting from depletion of CD30-expressing regulatory T-cells, highlighting the complexity of the therapeutic response beyond simple antigen density [112]. The success of brentuximab vedotin validated the antibody–drug conjugate (ADC) approach as a strategy to overcome the limited efficacy observed with unconjugated antibodies, encouraging the development of ADCs directed against additional lymphoma-associated targets. Among these, camidanlumab tesirine (ADCT-301) represents an emerging CD25-directed ADC composed of a human anti-CD25 mAb linked to the pyrrolobenzodiazepine (PBD) dimer SG3199. Following CD25-mediated delivery, the released PBD payload induces DNA minor groove binding and interstrand cross-link formation, leading to replication blockade and cell death [121]. Another promising antigen-directed strategy targets CC chemokine receptor 4 (CCR4), which is frequently expressed in subsets of PTCL, including AITL. Mogamulizumab is a glycoengineered, defucosylated humanized anti-CCR4 mAb that has shown encouraging activity in a multicenter phase II study, with an overall response rate of approximately 50% reported in patients with AITL [122]. In addition, a retrospective analysis suggested improved 4-year overall survival when combined mogamulizumab and chemotherapy treatment was compared with chemotherapy alone (46.3% vs. 20.6%) [123].

Alemtuzumab represented one of the earliest attempts to introduce antibody-based immunotherapy in PTCL by targeting the CD52 antigen (Figure 3A). Initial studies reported encouraging response rates, but the broad expression of CD52 on normal mature lymphocytes resulted in profound and prolonged lymphocyte depletion. Consequently, the clinical benefit of alemtuzumab was largely counterbalanced by severe immunosuppression, leading to opportunistic infections and treatment-related complications that limited its widespread application [124]. This experience highlights one of the major challenges of immunotherapy in T-cell lymphomas: the difficulty of identifying tumor-specific antigens, as many potential targets are shared between malignant and healthy T-cell populations.

Beyond conventional monoclonal antibodies and antibody–drug conjugates, bispecific antibodies (BsAbs) have emerged as a novel strategy to actively redirect immune effector cells toward malignant targets. In contrast with traditional antibodies, these molecules simultaneously interact with tumor-associated antigens and immune effector receptors, enhancing anti-cancer activity (Figure 3B) [112]. AFM13 is a first-in-class tetravalent bispecific innate cell engager that targets CD30 on lymphoma cells and CD16a on natural killer cells and macrophages, resulting in concomitant antibody-dependent cellular cytotoxicity and phagocytosis [125]. In the phase II REDIRECT trial, AFM13 showed promising activity in R/R CD30-positive PTCL, with the highest response rates observed in the AITL subgroup (ORR 53.3%; CRR 26.7%; [126]). Building on the activity of AFM13, the ongoing phase II LuminICE-203 trial evaluated AFM13 in combination with the allogeneic cord blood-derived NK cell product (AB-101) in patients with R/R CD30-positive lymphomas, including PTCL subtypes. This approach exploits AFM13-mediated CD30/CD16A engagement to enhance NK cell-mediated cytotoxicity. However, further clinical development of AFM13 was discontinued following the dissolution of Affimed, the company developing the drug, and the drug is no longer clinically available.

These findings suggest that immune-cell engaging strategies may be particularly effective in AITL; however, further studies are required to establish its optimal therapeutic positioning. In line with that, although AITL originates from malignant Tfh cells, it often features prominent interactions with B-cell infiltrates [110,127]. This prompted the exploration of B-cell–targeted therapy, in an attempt to attenuate the malignant phenotype. Rituximab is a human/murine chimeric anti-CD20 mAb, and since its first approval, it has revolutionized the treatment of B-cell malignancies [128]. A recent phase II Chinese study evaluating a triplet regimen of rituximab, lenalidomide, and the HDAC inhibitor, chidamide, in R/R AITL reported an impressive ORR of 71.4%, with a complete response rate of 32.1%, median PFS of 5.5 months, and a manageable toxicity profile [129].

Given the prominent role of immune dysregulation and the limitations associated with targeting antigens shared between malignant and normal T-cells, therapeutic strategies aimed at reactivating antitumor immunity have gained increasing interest in AITL. Immune-modulating antibodies target inhibitory pathways within the tumor microenvironment, with the goal of restoring immune effector function and enhancing endogenous responses against lymphoma cells [130]. Immune checkpoint inhibitors targeting the PD-1/PDL1 axis have been investigated in PTCL, although their role remains controversial in AITL. PD-1 is physiologically expressed at high levels on Tfh neoplastic cells and contributes to the regulation of germinal center immune responses. In the largest prospective phase II study conducted in R/R PTCL, the anti-PD-1 mAb geptanolimab achieved an ORR of 40.4% and a CR of 14.6% [131]. However, encouraging activity has not translated into consistent clinical benefit across PTCL subtypes. Studies with nivolumab [132] and pembrolizumab [133] have reported only modest efficacy. In particular, hyper-progressive disease was observed in several AITL patients treated with nivolumab [132], raising concerns that PD-1 may function as a tumor suppressor in certain T-cell lymphomas rather than solely as an inhibitory immune checkpoint [134]. In summary, PD-1 blockade in AITL is still poorly defined, and treatments in combination with anti-PD-1 antibodies are, at best, evaluated in early trials or in preclinical settings; therefore, caution for clinical application of these drugs is warranted. Similarly, inducible T-cell co-stimulator (ICOS) is a costimulatory receptor selectively expressed at high levels on Tfh cells, where it regulates their differentiation, survival and interaction with B-cells within germinal centers. MEDI-570 is a fully human mAb targeting ICOS, designed to induce depletion of ICOS-expressing cells through antibody-dependent cellular cytotoxicity. In the first-in-human phase I NCI-9930 trial, MEDI-570 was assessed in patients with R/R Tfh-like PTCL, including AITL. Treatment resulted in sustained reductions in circulating CD4^+^ICOS^+^ T-cells and demonstrated preliminary clinical activity, with an overall response rate of 44% in AITL patients, the largest subgroup of PTCL in this trial, with 12 out of a total of 17 patients. Although further validation is required, ICOS-directed therapy represents a biologically driven approach that exploits lineage dependency instead of merely targeting a tumor-associated surface antigen [135].

#### 4.4.2. Chimeric Antigen Receptor—T-Cell Therapies for T-Cell Lymphoma

**Figure 4:**
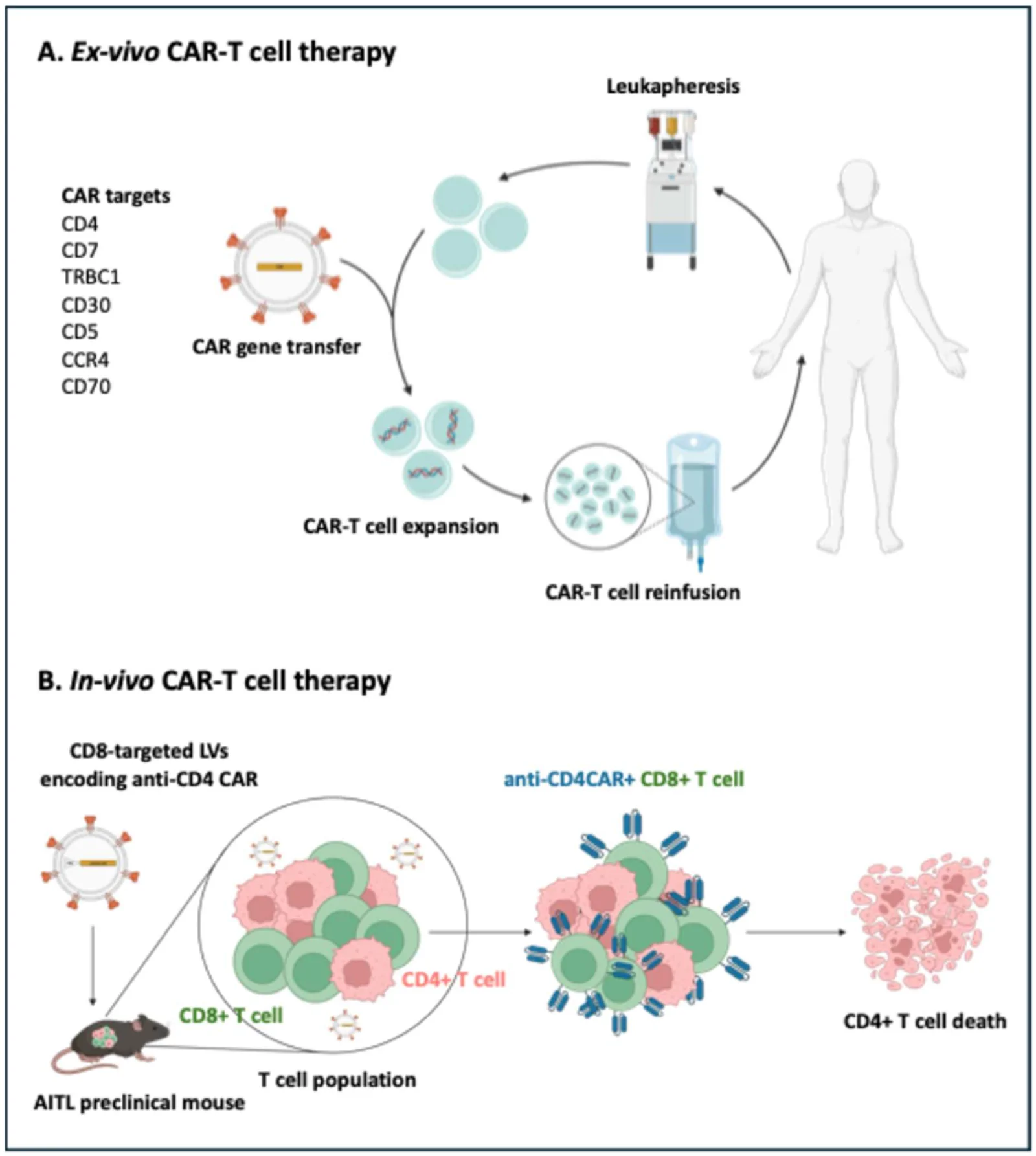
**Ex vivo and in vivo CAR T-cell therapy for peripheral T-cell lymphoma.** (**A**) Ex vivo CAR T-cell therapy. Blood is taken from patients, and T-cells are isolated; then they are stimulated and transduced with a lentiviral vector encoding a CAR directed against the malignant T-cells (CAR targets are indicated). Then follows the expansion of the CAR-expressing T-cells before their re-infusion into the patient who underwent some lymphocyte-depleting conditioning. (**B**) In vivo CAR T-cell therapy. Here, the lentiviral vector, which targets CD8 T-cells for in vivo gene transfer, will generate in vivo CD8 T-cells expressing an anti-CD4CAR directed against neoplastic AITL and healthy T-cells. Created in BioRender. Legros, V. (2026) https://core.local.biorender.dev/api/short-link/7ochj12.

Chimeric antigen receptor (CAR)-T-cell therapy represents a major advancement in cancer immunotherapy. It is an adoptive cell therapy in which autologous or allogeneic T lymphocytes are genetically engineered to express synthetic receptors capable of recognizing predefined cell-surface antigens (Figure 4A). Upon antigen engagement, CAR-T-cells become activated, proliferate, and mediate targeted cytotoxicity against malignant cells [136]. Following the remarkable clinical success of CD19-directed CAR-T-cell therapy in B-cell leukemias and lymphomas, considerable interest has emerged in extending this strategy to peripheral T-cell lymphomas, including AITL. However, the application of CAR-T therapy for T-cell malignancies poses unique biological and technical challenges. Indeed, prolonged T-cell aplasia after treatment, the contamination of autologous CAR-T products with malignant T-cells, and the risk of fratricide during CAR-T manufacturing, in which CAR-T-cells inadvertently kill one another due to shared target antigen expression, present major challenges. Consequently, the generation of CAR-T therapies for AITL has largely remained at the preclinical stage, with current efforts focusing on the identification of lineage-associated targets and the engineering of strategies to improve specificity and safety [137].

One of the earliest proof-of-concept studies explored CD4 as a lineage-associated target for CAR T-cell therapy, supported by previous clinical experience with CD4-directed antibodies such as zanolimumab (HuMax-CD4), which established CD4 as a therapeutically accessible target in T-cell malignancies [138]. Pinz et al. [139,140] engineered CD4-directed CAR-T-cells and demonstrated potent cytotoxic activity against CD4-positive T-cell lymphoma cell lines and primary patient samples, together with significant antitumor efficacy in xenograft mouse models. More recently, Krug et al. [141] extended this concept by developing an in vivo CAR T-cell generation strategy specifically designed for Tfh-like PTCL (Figure 4B). Instead of manufacturing CAR-T-cells ex vivo, the authors employed CD8-targeted lentiviral vectors (LVs) to selectively transduce endogenous CD8 T-cells, enabling in situ generation of anti-CD4 CAR-T-cells. In a murine model of AITL [42], this approach efficiently eliminated malignant Tfh cells and significantly prolonged AITL mouse survival [141]. By restricting CAR expression to CD8 T-cells, this strategy also minimized fratricide during CAR generation, illustrating how delivery platform engineering may help overcome some of the unique challenges associated with CAR-T-cell therapy for T-cell malignancies. CD7 represents another example of the challenges associated with targeting shared T-cell lineage antigens. Although broadly expressed across malignant T-cell populations, CD7 is also present on normal T-cells, leading to CAR-T fratricide during manufacturing. To overcome this limitation, CRISPR/Cas9-mediated disruption of CD7 has been explored to generate fratricide-resistant CD7 CAR-T-cells with preserved antitumor activity [142]. However, the therapeutic relevance of CD7 targeting in AITL remains uncertain, as Tfh-like PTCLs display heterogeneous CD7 expression.

Rather than eliminating a shared lineage marker, TRBC1-directed CAR-T therapy exploits the clonotypic nature of mature T-cell malignancies by targeting a TCR β-chain constant region selectively expressed by the malignant clone. The rationale behind this approach derives from the unique organization of the T-cell receptor (TCR) β-chain constant region. Two mutually exclusive constant domains, TRBC1 and TRBC2, are expressed during T-cell development, resulting in a physiological T-cell repertoire composed of both populations. In contrast, malignant T-cell lymphomas arise from the expansion of a single transformed clone and therefore express only one TRBC variant. Consequently, targeting the TRBC isoform expressed by the malignant clone allows preferential elimination of lymphoma cells while sparing normal T-cells expressing the alternative isoform, thereby preserving part of the endogenous T-cell repertoire [143]. Based on this concept, AUTO4 was developed as an ex vivo autologous TRBC1-directed CAR-T-cell product. To enable the generation of functional TRBC1-specific CAR-T-cells, the manufacturing process includes depletion of endogenous TRBC1-positive T-cells prior to genetic modification, thus preventing fratricide during CAR-T-cell expansion. The phase I/II LibraT1 trial (NCT03590574) evaluated AUTO4 in patients with R/R TRBC1-positive PTCL, including AITL. However, this was a small cohort including five Tfh-like PTCL patients and four AITL patients, which indicated that the efficacy results should be interpreted with caution and not generalized. Preliminary results demonstrated the feasibility and tolerability of this approach, with limited severe immune toxicity and selective targeting of TRBC1-positive malignant populations while preserving TRBC2-positive T-cells [144]. Although longer follow-up and larger cohorts are required, these findings provide the first clinical evidence that clonality-based antigen targeting may represent a viable strategy for CAR T-cell therapy in T-cell malignancies.

Given the established clinical relevance of CD30 as a therapeutic target in PTCL, CD30-directed CAR T-cell therapy has also been explored as a potential strategy for relapsed or refractory disease. Early clinical studies evaluating autologous CD30 CAR-T-cells have demonstrated feasibility and preliminary antitumor activity in CD30-positive lymphomas; however, experience in PTCL remains limited, and the specific efficacy in AITL remains difficult to define due to the small number of treated patients [145,146]. The search for additional therapeutic targets is ongoing, with approaches directed against other lineage-associated or tumor-associated antigens, including CD5, CCR4 and CD70. However, their translation into AITL remains challenging due to heterogeneous expression patterns and the potential overlap with normal T-cell populations [112].

### 4.5. Metabolic Changes in AITL Might Point to New Targets for Treatment

#### 4.5.1. Cancer and Metabolism

Metabolic reprogramming of cancer cells has now been put forward as one of the cancer hallmarks that promote cancer progression by driving cell proliferation and survival but also therapeutic resistance [147,148]. Two major metabolic pathways, glycolysis and oxidative phosphorylation (OXPHOS), produce adenosine triphosphate (ATP) to supply the energy required by each cell. Glycolysis is used by multiple cell types to generate energy for proliferation and growth. OXPHOS, or mitochondrial respiration, is the energy bank of the cell because it produces much more ATP as compared to glycolysis (36 ATP for OXPHOS versus 4 ATP for glycolysis [149]). In unstressed conditions, healthy cells balance nutrient consumption and metabolism to remain functionally competent and maintain the ability to divide [150,151,152]. In contrast, cancer cells frequently reprogram their metabolism to adapt to their microenvironment and to satisfy their high energy needs. Cancer cells often utilize glucose via aerobic glycolysis, also called the Warburg Effect [151,152]. However, this view is now somewhat outdated, and the complex metabolic reprogramming that cancer cells use to cope with harsh environmental conditions is now better understood. It is now accepted that reprogramming toward OXPHOS metabolism frequently occurs in tumors [150,153,154]. Importantly, many of these metabolic switches are also orchestrated by co-inhibitory pathways, e.g., the PD-1/PD-L1 axis engaged by tumor cells in order to impede immune cell functions [155]. Therefore, identifying and targeting the specific immune-metabolic modifiers might have significant clinical implications in cancers where co-inhibitory signals play a major role. This is the reason why current research is focusing on the identification of the metabolic requirements of cancer cells, particularly in the context of T-cell lymphoma might reveal new therapeutic opportunities and first studies in this context are reported below.

#### 4.5.2. Identification of Neoplastic AITL T-Cell Metabolism and New Treatment Options

**Figure 5:**
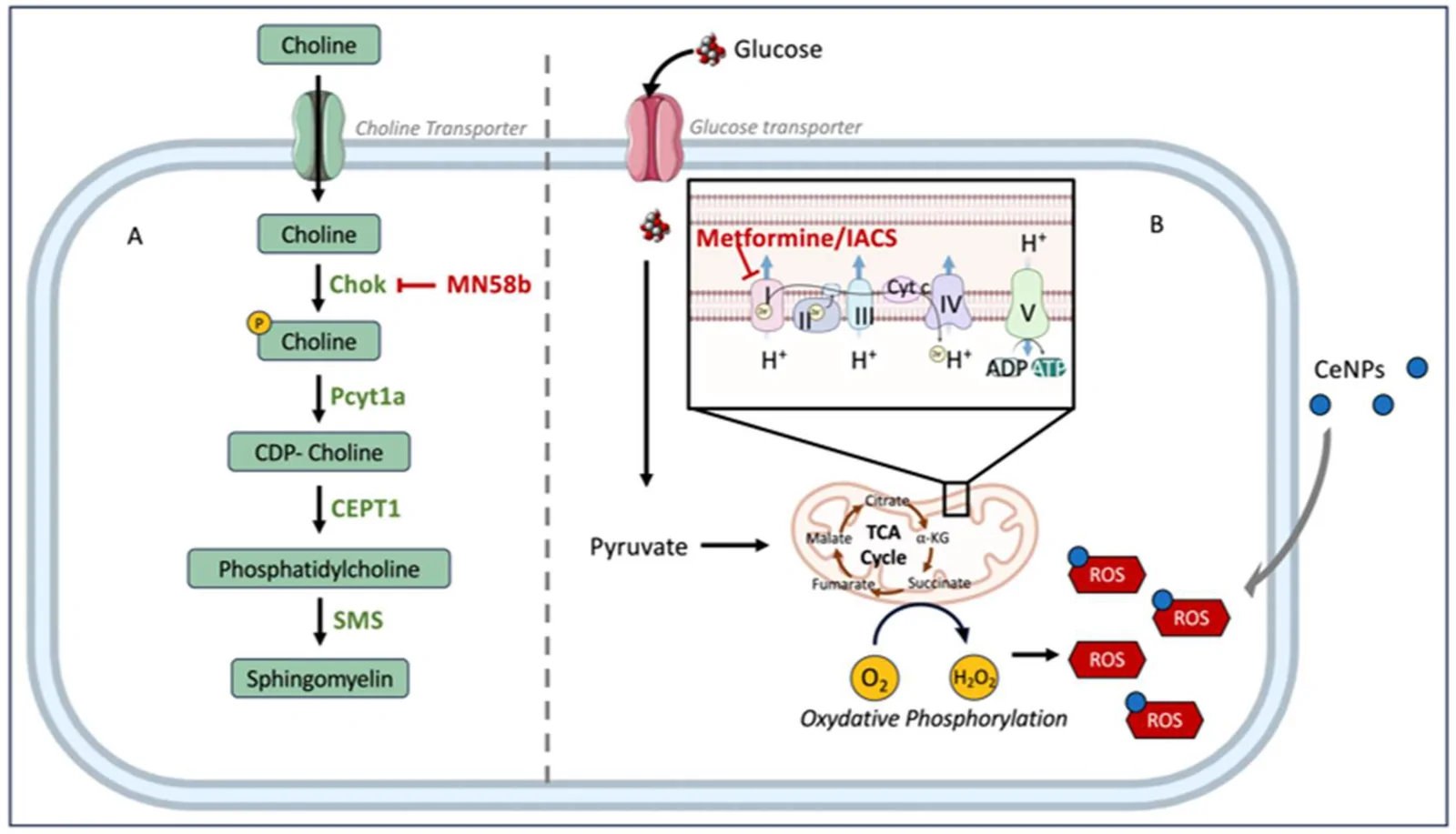
**AITL addiction to metabolic pathways uncovers new therapeutic options.** (**A**) Schematic representation of the CDP-choline pathway, which is overactivated in AITL malignant T-cells. Inhibition of one of the enzymes in the pathway (CHOKA) by the drug MN58b was shown to increase survival in a preclinical model for AITL [174]. (**B**) Representation of mitochondrial respiration, which is augmented in AILT CD4 T-cells. The complexes I, II, III, IV, and V form the respiratory chain in the mitochondria. Metformin and the drug IACS are inhibitors of complex I. The consequence of this high oxidative respiration is the high release of reactive oxygen species (ROS), which were shown to be inhibited by Cerium nanoparticles (CeNPs; [172]).

In 2024, Krug et al. [156] were able to determine that human AITL malignant T-cells are dependent on mitochondrial respiration (Figure 5). It was confirmed in their preclinical AITL mouse model that neoplastic cells use primarily the OXPHOS pathway for their energy needs, which allowed them to evaluate electron transport chain (ETC) inhibitors. Metformin, for instance, an inhibitor of complex I of the ETC [157,158], not only increased the survival of AITL mice but also induced the reactivation of the cytotoxicity of CD8 cells against malignant cells present in the spleen or the lymph nodes of these AITL mice (Figure 5B). Yet, metformin can also have off-target effects, which represent a safety risk. Therefore, these results were confirmed employing another, more specific Complex I inhibitor (IACS-010759; [159,160]). The therapeutic effect on the AITL mouse model was significant at doses that did not show toxicity in a Phase I clinical trial [161]. Mitochondrial respiration in malignant CD4^+^ PD-1^high^ cells was significantly inhibited by IACS, leading to their elimination. Additionally, consistent with these results in the AITL mouse model, a retrospective study including a cohort of diabetic PTCL patients showed increased overall survival and progression-free survival upon treatment with metformin [162]. To understand the reason why these tumoral cells rely more on mitochondrial metabolism, we can assume that malignant cells have a Tfh phenotype and are found in the “B zones” of lymphoid organs. These cells are therefore found in close proximity to B lymphocytes in germinal centers, which largely use glycolysis as their energy source. T-cells therefore compete for access to glucose in the TME, which may explain their adaptation to mitochondrial metabolism [163,164]. Thus, temporarily targeting these B-cells in the AITL tumor context (e.g., anti-CD20 immunotherapy) might support existing or proposed therapeutic strategies [129,165]. Moreover, the PD-1 receptor present on the surface of tumor cells also plays a role. It is clear that both human and murine CD4 cells with high PD-1 expression are more dependent on mitochondrial respiration than cells with lower PD-1 expression in AITL. This correlates with the work of Patsoukis [166], which shows that PD-1 activation inhibits glucose uptake and glycolysis activation in favor of β-oxidation and lipolysis [166]. Furthermore, in the case of AITL, the binding of PD-1 to its ligand PDL1 is even more important since it binds malignant Tfh cells to B-cells in germinal centers, which support each other. This was confirmed by targeting PD-1 in mAITL mice, resulting in improved survival and reduced tumor growth in vivo [42]. In humans, encouraging results have been shown in clinical trials in patients with PTCL (excluding AITL) treated with geptanolimab, an anti-PD-1 monoclonal antibody. The combination of metformin and an anti-PD-1 antibody was tested in the AITL preclinical model, as has been done for other solid cancers such as melanoma and colon cancer [156,167], but it showed no beneficial effect, and mice treated with this combination did not show better survival than control mice.

A consequence of strong electron transport chain activity is the production of excessive ROS in growing tumor cells that become addicted to high reactive oxygen species (ROS) levels [168,169,170,171]. Indeed, in a mAITL preclinical mouse model, it was demonstrated that the malignant Tfh T-cells showed high ROS levels as compared to healthy counterpart CD4 T-cells and CD8 T-cells. Moreover, the exhausted CD8 T-cells in the AITL were also low in ROS levels. Therefore, Krug et al. [172] proposed targeting malignant cell metabolism by scavenging ROS in AITL cells (Figure 5B). One of the most powerful ROS scavengers is CeO_2_ nanoparticles [173]. They can neutralize ROS but will scavenge the ROS in excess in the cell and therefore not interfere with their role in immune functions of healthy cells. Administration of Cerium oxide (CeO_2_) nanoparticles in an AITL mouse model reduced ROS levels in the malignant T-cells. This led to AITL T-cells death because of their mitochondrial respiration/ROS addiction. A single CeO_2_ nanoparticle administration increased the survival of mAITL mice by a fold. Impressively, the CeO_2_ Nanoparticles restored ROS balance in the tumoral microenvironment, since exhausted CD8 T infiltrating lymphocytes were reversed to functional cytotoxic T-cells [172].

#### 4.5.3. Altered Lipid Metabolism in AITL Reveals New Treatment Possibilities

In an attempt to identify a specific metabolic pathway addiction in neoplastic AITL Tfh cells, Krug et al. [174] performed an integrated analysis of proteomic and transcriptomic data comparing murine CD4 Tfh AITL cells from the plck-GAPH model (Figure 1) with healthy murine CD4 T-cells. They showed that tumoral cells exhibit increased lipid metabolism as compared to their healthy counterparts (Figure 5A). Moreover, this was confirmed by gene expression analysis in human AITL malignant T-cells. Therefore, initially, a global inhibition of lipid metabolism by the carnitine palmitoyltransférase 1 (CPT1) inhibitor, etomoxir, was evaluated and increased the survival of a preclinical AITL mouse model. Furthermore, it has been shown that etomoxir not only interferes with β-oxidation, but at higher concentrations (>100 µM) it can inhibit mitochondrial respiration by blocking complex I of the mitochondrial respiratory chain [175,176]. Yet, it is possible to affect T malignant cells with a concentration of etomoxir at 30 μM that theoretically does not inhibit the Electron Transport Chain [177]. It is not a surprise to see a potent effect of etomoxir at high doses (300 μM) since AITL cells rely a lot on OXPHOS metabolism for their survival. At high doses, etomoxir might be as efficient in targeting mitochondrial metabolism as in targeting lipid metabolism [174].

A more detailed analysis of the lipid pathways involved identified the choline lipid pathway as being strongly active in murine AITL cells, which was again confirmed for human AITL (Figure 5B). Phosphatidyl choline, the final product of this pathway, is a major lipid component of the cell membrane and plays a role in mitosis and cell signaling. To target the increased choline metabolism detected in AITL neoplastic cells, it has been proposed to inhibit this lipid pathway at its first step using the choline kinase A (CHOKA) inhibitor MN58b. Krug et al. confirmed a decrease in T-cell lymphoma development following MN58b administration in an AITL preclinical mouse model. However, prolonged in vivo administration of MN58b proved to be toxic for the recipient mAITL mice. [174]. With the same objective to inhibit choline metabolism, a CHOKA inhibitor, TCD-717, has been tested in a phase I trial in patients with solid tumors, but the results are not available, which unfortunately prevents us from having an idea of the efficacy and safety of this treatment. It is clear, though, that more efficient and better tolerated CHOKA inhibitors are needed and are the subject of particular research focus [178].

The accumulation of phosphatidyl choline (PC) can be found in many different cancers [179,180]. In addition to the Kennedy pathway, in the case of AITL, PC accumulation could be partly attributed to another lipid production pathway: the Lands cycle. This cycle involves the conversion of lysophosphatidylcholine (LPC) to PC via lysophosphatidylcholine acyltransferase 1 (LPCAT1). Modulation of LPCAT1 may therefore play an important role in the pathogenesis and progression of cancer [181,182], and we have found that this enzyme is also significantly upregulated in Tfh cells in human and murine AITL, suggesting that inhibition of LPCAT1 could also be a new therapeutic option, but this still has to be evaluated. A promising candidate that could be used as a specific non-competitive inhibitor of LPCAT is 5-Hydroxyethyl 5,3ʹ thiophenyl pyridine (HETP). In the cellular systems used, HETP was found to have a median inhibitory concentration (IC50) of 10μM for LPCAT inhibition. It could therefore be a viable option for the treatment of AITL if no severe toxicity is detected during in vivo administration [183].

In addition, phospholipases D (PLDs) form a superfamily of enzymes responsible for the hydrolysis of phosphatidylcholine into choline and phosphatidic acid (PA), a lipid signaling molecule. Among this family are PLD1 and PLD2, which are overexpressed in AITL, and PLDs are involved in the progression of cancers via the activation of several PA-dependent pathways [184]. Recently, a highly efficient and specific PLD1 inhibitor was developed using computer-assisted design. The inhibitor promoted apoptosis in colorectal cancer cells, but not in normal colon cells. PLD1 inhibition also enhanced the immune response of macrophages and cytotoxic T-cells against cancer cells [185]. This is an active area of research with new PLD inhibitors being discovered and developed, which may offer prospects for the treatment of AITL.

## 5. Conclusions and Perspectives

Several aspects of AITL and Tfh-like PTCL disease explain the difficulties in finding an adapted treatment: (1) the late detection of the lymphoma; (2) the complex mutational landscape, which leads to subgrouping of patients to allow specific treatment and move away from current chemotherapy; (3) the strong immunosuppressive and tumor-supporting environment; (4) the rareness of AITL, which makes evaluation of treatments still difficult, though lately valid animal models offer help in this respect. Clearly, the better results are obtained by combination therapies (chemo- and immunotherapy, simultaneous epigenetic interference using HDACi and DNA demethylating agent), but we need to consider the toxicity and safety. The metabolic inhibitors proposed for AITL/PTCL although effective might in human at affective dose proof to be too toxic. These novel treatments proposed for AITL patients, however, still need thorough evaluation in clinical trials, and it might be advised to subgroup patients according to their genetic mutational combinations to orient them to one or a combination of more treatment options to reach a significant benefit for the patient. Another focus in the future for this disease might be to compete with the hostile TME, which might allow other therapies such as antibody-based therapies or CAR T-cell therapies to be more efficient. More recently, there is awareness of suppressive subtypes of tumor-associated macrophages (TAMs) in tumors. Therapeutic strategies rely on avoiding the recruitment of macrophages or disrupting their survival in the TME, or reprogramming macrophages to change their phenotype in order to enhance their apoptosis [186,187].

Immunotherapies have substantially improved the therapeutic landscape of AITL by providing more selective and potent antitumor strategies than conventional chemotherapy. Nonetheless, their clinical efficacy remains constrained by the complex biology of the disease, as antigen heterogeneity, the lack of truly tumor-specific targets and the profound immune dysregulation that characterizes the AITL microenvironment. For antibody-based therapies, future progress will largely depend on the identification of more selective therapeutic targets and on the development of next-generation antibody formats capable of simultaneously enhancing antitumor activity and modulating the tumor immune microenvironment.

These challenges are even more relevant for CAR-T-cell therapy, where target selection must be integrated with strategies to improve safety, manufacturing and clinical accessibility. Novel engineering approaches, including adaptor CAR platforms, which enable reversible and modular control of CAR-T-cell activity, may improve treatment safety while increasing flexibility and reducing on-target/off-tumor toxicity [188,189]. In addition, improving the feasibility and accessibility of CAR-T therapy represents another major challenge. Conventional autologous CAR-T manufacturing requires patient cell collection, ex vivo genetic modification, expansion and extensive quality control, resulting in complex logistics, prolonged production times and high costs. In this context, in vivo CAR-T generation strategies represent a promising alternative by allowing direct reprogramming of endogenous T-cells within the patient [190,191]. Recently, a first-in-human trial was reported successful for in vivo CAR T-cells generation in patients suffering from multiple myeloma, though not without side effects [192]. Although challenges related to selective delivery, safety and long-term persistence remain, targeted in vivo CAR therapy represents an innovative direction that could expand the applicability of cellular immunotherapies in AITL and other T-cell malignancies. All the novel treatments evaluated in clinical trials are still not curative, though important progress has been made in terms of overall survival and progression-free survival in AITL patients. Importantly, gaining time using these new drugs might allow a better chance to find a compatible allogenic HSC donor. Side effects of these novel treatments are also strongly reduced as compared to classical chemotherapy, which might also allow a better context to perform autologous salvage HSC engraftment and increase its success. In vivo CAR-T-cell therapy could, once the estimated 10-fold reduction in costs is confirmed, be used as first-line treatment in AITL. Moreover, immediate off-the-shelf accessibility might be essential for patients with this kind of hyper-aggressive T-cell lymphoma. On the one hand, immunotherapies in general (also antibody-based) are not as toxic as current first-line chemotherapy because of their higher specificity for the tumor target cells. On the other hand, interventions with epigenetic or metabolic interfering drugs that are not specific enough for the malignant cells might cause toxicity in healthy cells. In a particular case, though, where the inhibitors are highly specific for one of the mutations found in AITL, important off-target effects are not expected. For example, Enasidenib, a selective epigenome-interfering drug, specifically targets tumor cells carrying *IDH2* mutations. Summarizing, although progress in AITL and PTCL treatments were achieved, the search for better effective and possible curative treatments is still open.

## Data Availability

No new data were created or analyzed in this study. Data sharing is not applicable to this review.

## Abbreviations

The following abbreviations are used in this manuscript:

AITL	Angio-immunoblastic T-cell lymphoma
aKG	a-ketoglutarate
allo-HCT	allogeneic hematopoietic cell transplantation
ADC	antibody–drug conjugate
AP1	activator protein 1
ASCT	autologous stem-cell transplantation
Bcl-6	B-cell lymphoma 6
BM	bone marrow
BV	Brentuximab vedotin
BsAb	bispecific antibodies
BTK	bruton tyrosine kinase
A+CHP	BV plus cyclophosphamide, doxorubicin and prednisone
CAR T	chimeric antigen receptor T-cell
CARD11	a scaffold protein that organizes signaling complexes essential for adaptive immunity
Cas9	CRISPR-associated protein 9
CCL17	CC chemokine ligand 17
CCR4	C-C chemokine receptor type 4
CeO_2_	cerium oxide
CHOEP	etoposide +CHOP
CHOKA	choline kinase a
CHOP	cyclophosphamide + doxorubicin + vincristine + prednisolone chemo regiment
CPT1	carnitine palmitoyltransférase 1
CR	complete response
CREBBP	CREB binding lysine acetyltransferase
CRISPR	clustered regularly interspaced short palindromic repeats
CTNNB1	Catenin Beta 1
CXCL13	C-X-C motif chemokine ligand 13
CXCR5	C-X-C motif chemokine receptor 5
D2HG	D-2-hydroxyglutarate
DNMT3	DNA methyltransferase
EP300	EP300 Lysine Acetyltransferase
ETC	electron transport chain
EZH2	Enhancer Of Zeste 2 Polycomb Repressive Complex 2 Subunit
Fas	Fas Cell Surface Death Receptor
FDA	U.S. Food and Drug Administration
FDC	follicular dendritic cells
FER	FER tyrosine kinase
FYN	FYN proto-oncogene, Src family tyrosine kinase
GAPDH	glyceraldehyde 3-phosphate dehydrogenase
GC	germinal center
GSEA	gene set enrichment analysis
GTPase	hydrolase enzymes that bind to the nucleotide guanosine triphosphate (GTP) and hydrolyze it to guanosine diphosphate (GDP).
HDACi	histone deacetylase inhibitor
HETP	5-Hydroxyethyl 5,3′ thiophenyl pyridine
IC	immune checkpoint
IC50	median inhibitory concentration
ICOS	Inducible T-cell costimulator
IDH2	isocitrate dehydrogenase-2
IKK	IκB kinase
IL-6	interleukin 6
ITK	IL2 Inducible T-Cell Kinase
JAK1/2	janus kinase 1/2
KDM6A	gene Lysine Demethylase 6A
KMT2D	lysine Methyltransferase 2D
LPC	lysophosphatidylcholine
LPCAT1	lysophosphatidylcholine acyltransferase 1
MAPK	mitogen-activated protein kinase
MMAE	microtubule-destroying agent monomethyl auristatin E
mAbs	monoclonal antibodies
mTOR	mechanistic target of rapamycin
NEMO	subunit of the IκB kinase complex that activates NF-κB
NF-κB	nuclear factor kappa-light-chain-enhancer of activated B-cells
NFAT	nuclear factor of activated T-cells activator protein 1
NFAT	nuclear factor of activated T-cells
NIK	NF-κB-inducing Kinase
NK	natural killer cell
NP	nanoparticle
ORR	objective response rate
OS	overall survival
OT-II mice	a genetically engineered, transgenic mouse strain widely used in immunology research to study CD4^+^ helper T-cell responses
OXPHOS	oxidative phosphorylation
PA	phosphatidic acid
PC	phosphatidyl choline
PD-1	programmed cell death protein 1
PDL1	programmed death-ligand 1
PFS	progression-free survival
PI3K	phosphatidylinositol 3-kinase
PLCG1	phospholipase C gamma 1
PLD	phospholipases D
PTCL-NOS	peripheral T-cell lymphoma not otherwise specified
PTCL	peripheral T-cell lymphoma
R/R	relapsed or refractory
RHOA	ras homolog family member A
ROS	reactive oxygen species
STAT	signal transducers and activators of transcription
SYK	Associated Tyrosine Kinase
TBK1	TANK-binding kinase 1
TCR	T-cell receptor
TET2	ten-eleven translocation-2
Tfh	follicular helper T-cells
TIL	tumor-infiltrating lymphocytes
TME	tumor microenvironment
TRBC1	T-cell receptor (TCR) β-chain constant region 1
VAV1	vav guanine nucleotide exchange factor 1
VEGF	vascular endothelial growth factor
WHO	World Health Organization
